# Strategies to improve adherence to exercise-based phase II cardiac rehabilitation after percutaneous coronary intervention: a best evidence summary

**DOI:** 10.3389/fcvm.2026.1868542

**Published:** 2026-07-15

**Authors:** Hao Guo, Chengyu Xia, Yuheng Du, Liuxia Ji, Xuehui Yang, Hui Liu

**Affiliations:** Department of Cardiology, The Second Affiliated Hospital of Shantou University Medical College, Shantou, Guangdong, China

**Keywords:** cardiac rehabilitation, exercise adherence, percutaneous coronary intervention, phase II cardiac rehabilitation, summary of the evidence

## Abstract

**Objective:**

To identify, appraise, and synthesize the best available evidence on strategies to improve adherence to exercise-based phase II cardiac rehabilitation in patients after percutaneous coronary intervention, and to provide evidence-informed guidance for clinical practice.

**Methods:**

Guided by the 6S evidence pyramid, a systematic search was conducted across evidence-based resources, guideline repositories, professional association websites, and primary literature databases for studies addressing strategies to improve adherence to exercise-based phase II cardiac rehabilitation after percutaneous coronary intervention. The search covered the period from database inception to November 30, 2025. Two reviewers independently screened the literature, appraised methodological quality using design-specific tools, and synthesized the evidence according to the Joanna Briggs Institute evidence grading and recommendation system.

**Results:**

A total of 24 studies were included, comprising 1 clinical decision support resource, 6 guidelines, 6 systematic reviews and meta-analyses, 6 expert consensus statements, 4 randomized controlled trials, and 1 quasi-experimental study. Thirty-two evidence statements were synthesized across seven dimensions: program referral and initiation, multidisciplinary cardiac rehabilitation team, individualized exercise prescription, adherence-enhancing interventions, population-specific strategies, social support, and quality management and outcome evaluation.

**Conclusion:**

This study synthesized the best available evidence on strategies to improve adherence to exercise-based phase II cardiac rehabilitation in patients after percutaneous coronary intervention. The findings provide an evidence-informed basis for clinical practice and may support healthcare professionals in developing context-sensitive rehabilitation strategies to enhance patient participation and sustained engagement in phase II cardiac rehabilitation.

**Systematic Review Registration:**

Evidence-Based Nursing Center of Fudan University. Registration number ES20257186.

## 1 Introduction

Coronary heart disease (CHD) is a common cardiovascular disease caused by coronary atherosclerosis, which can lead to narrowing or even occlusion of the coronary arteries, thereby resulting in myocardial ischemia, hypoxia, or necrosis (1, 2). Percutaneous coronary intervention (PCI), as an important coronary revascularization strategy, has been widely used in the clinical treatment of CHD and can relieve ischemic symptoms by alleviating coronary stenosis or occlusion and improving myocardial perfusion (3). However, although PCI can effectively restore coronary blood flow, many patients still face challenges after the procedure, including recurrent angina, functional limitations, psychosocial problems, and reduced quality of life. Therefore, scientifically grounded rehabilitation management for patients after PCI is of considerable clinical importance (4).

Exercise-based cardiac rehabilitation (CR) is a multidisciplinary, comprehensive rehabilitation model centered on exercise training that aims to promote physical, psychological, and social recovery in patients with cardiovascular disease (5). According to the stage of implementation, cardiac rehabilitation is generally divided into Phase I inpatient rehabilitation, phase II early outpatient rehabilitation, and phase III long-term maintenance rehabilitation (6). With the shortening of hospital stays after PCI, the focus of rehabilitation has gradually shifted from the inpatient setting to the post-discharge period (7). As a continuation of phase I inpatient rehabilitation and a bridge to phase III long-term maintenance rehabilitation, phase II cardiac rehabilitation is a critical stage for helping patients establish regular exercise behavior, sustain engagement in rehabilitation, and achieve ongoing recovery (6, 7). Previous studies have shown that exercise-based cardiac rehabilitation can reduce cardiovascular mortality and readmission while significantly improving quality of life, exercise capacity, and physical function (8). International guidelines have also recommended it as a core component of secondary prevention (9).

Despite the clear benefits of exercise-based cardiac rehabilitation, adherence among patients after PCI remains low. Studies from the United Kingdom, the United States, and China have shown that patients commonly experience low completion rates, insufficient sustained participation, and high dropout rates in exercise-based cardiac rehabilitation programs (10–12). A mixed-methods study by Xie et al. (13) identified multiple barriers to hospital-based phase II cardiac rehabilitation among patients with coronary heart disease, including practical barriers, limited social support, misconceptions about cardiac rehabilitation, psychological distress, and barriers related to rehabilitation programs and the healthcare system. These findings indicate that adherence to phase II cardiac rehabilitation is influenced by multiple factors involving patients, families, rehabilitation programs, and healthcare systems. Given the complexity of adherence, early identification of patients at risk of poor adherence is important. A scoping review by Xia et al. (14) summarized existing prediction models for adherence to cardiac rehabilitation programs among patients with cardiovascular disease and found that these models remain at an early stage of development and cannot yet be reliably recommended for routine clinical practice. This finding suggests that practical adherence-support strategies are still needed in clinical practice. Although major guidelines and position statements provide important recommendations for cardiac rehabilitation (15, 16), these documents often address cardiac rehabilitation broadly, rather than focusing specifically on adherence to exercise-based phase II cardiac rehabilitation after PCI. In addition, adherence-related strategies are scattered across guidelines, expert consensus statements, systematic reviews, randomized trials and other relevant original studies, which makes it difficult for multidisciplinary CR teams to identify and apply the most relevant evidence in practice. Therefore, this best-evidence summary was designed not simply to restate existing guideline recommendations, but to integrate fragmented evidence from multiple sources and translate it into concise, graded, and practice-oriented recommendations, thereby providing actionable guidance for improving adherence in this specific rehabilitation stage and patient population.

Evidence-based nursing is an approach to clinical decision-making that integrates the best available research evidence, clinical expertise, and patient preferences to support decision-making and improve health outcomes (17). Within the framework of evidence-based nursing, best evidence summaries can integrate the current best available evidence in a relatively concise and clinically accessible manner, thereby facilitating the translation of evidence into practice (17). Therefore, this study aimed to systematically synthesize the evidence related to improving adherence to exercise-based phase II cardiac rehabilitation in patients after PCI using an evidence-based approach, in order to provide a scientific basis for clinical practice.

## 2 Methods

### 2.1 Research design

This study adopted a best-evidence summary design. This approach is used to address a focused clinical question by systematically retrieving, critically appraising, and synthesizing existing evidence resources, and by translating the best available evidence into concise and practice-oriented recommendations (17, 18). It differs from conventional systematic reviews that primarily synthesize study-level evidence from original research, and from scoping reviews that mainly map the scope, characteristics, and gaps of an evidence field.

The methodological process was informed by the evidence-summary methodology of the Joanna Briggs Institute (JBI) (17). The process includes key components such as problem formulation, literature retrieval, study screening, critical appraisal, evidence synthesis, evidence grading, and recommendation formulation (17). To ensure reporting transparency and reproducibility, this study was reported with reference to the PRISMA 2020 statement where applicable (19). Although PRISMA 2020 is primarily a reporting guideline for systematic reviews, its core requirements for transparent reporting of the search process, study screening, eligibility assessment, and study-selection flow diagram are consistent with the reporting needs of this best-evidence summary. Therefore, PRISMA 2020 was used to guide reporting transparency, whereas the JBI evidence-summary methodology and evidence hierarchy were used to guide evidence appraisal, synthesis, grading, and recommendation formulation. The completed PRISMA 2020 checklist is provided in Supplementary Material S2.

This study was registered through the Evidence-Based Nursing Center of Fudan University (registration number: ES20257186). As this study was a secondary analysis of published literature, ethical approval was not required.

### 2.2 Problem establishment

The clinical question was formulated using the PIPOST framework developed by the Center for Evidence-Based Nursing at Fudan University (20). PIPOST shares several core elements with PICOS, including the population, intervention, outcome, and eligible study or evidence types. However, PIPOST is tailored to best-evidence summaries by additionally specifying the professional users of the evidence and the setting for evidence application. These elements are important because best-evidence summaries aim not only to identify and appraise evidence, but also to support the translation of evidence into practice-oriented recommendations.

The PIPOST elements in this study were defined as follows:

1. The first P (population, i.e., the target population for evidence application) referred to post-PCI patients aged 18 years or older;
2. I (intervention) referred to strategies for improving adherence to exercise-based phase II cardiac rehabilitation;
3. The second P (professional, i.e., the target users of the evidence) included healthcare professionals such as physicians, nurses, rehabilitation specialists, and other relevant clinical staff;
4. O (outcome) referred to the adherence rate, defined as the degree to which participants adhered to the prescribed exercise regimen (21);
5. S (setting, i.e., the context in which the evidence was applied) referred to settings appropriate for Phase II CR, including hospitals, clinics, homes, and communities (22);
6. T (type of evidence) comprised clinical decisions, clinical guidelines, systematic reviews, expert consensus statements, best clinical practices, evidence summaries, and relevant original studies related to the topic of this study.

### 2.3 Literature search

A systematic literature search was conducted using the “6S” pyramid model, covering evidence-based resources, guideline repositories, and primary literature databases. The search sources included three categories:

1) Evidence-based knowledge repositories: UpToDate, BMJ Best Practice, and Joanna Briggs Institute (JBI);
2) Guideline repositories: Guidelines International Network (GIN), Scottish Intercollegiate Guidelines Network (SIGN), National Institute for Health and Care Excellence (NICE), and Yimaitong;
3) Primary databases: Embase, Web of Science, Cochrane Library, PubMed, Wiley, China National Knowledge Infrastructure (CNKI), VIP, Wanfang Database, and Chinese Biomedical Literature Database (CBM).

In addition, the official websites of the European Society of Cardiology (ESC), American Heart Association (AHA), and American College of Cardiology (ACC) were systematically searched. All databases and resources were searched from inception to November 30, 2025, for eligible studies published in either English or Chinese. To ensure comprehensiveness, the reference lists of the included studies were also manually screened for additional relevant literature. The detailed search strategy, including database-specific search terms and Boolean operators, is provided in Supplementary Material S1.

### 2.4 Inclusion and exclusion criteria

The inclusion criteria were as follows: (1) post-PCI patients aged over 18 years; (2) studies addressing interventions to improve adherence to exercise-based phase II CR after PCI. (3) eligible literature types including clinical decisions, systematic reviews, guidelines, expert consensus statements, evidence summaries, and original studies; and (4) publications in Chinese or English.

Based on current phase-based descriptions of CR (15, 23–26), phase II CR in this review was considered as structured, professionally supported early post-discharge rehabilitation delivered in centre-based, outpatient, home-based, hybrid, or tele-rehabilitation formats after hospital discharge and before long-term maintenance rehabilitation. For studies that spanned both phase II and phase III CR or included transition-to-maintenance components, we included them only when the phase II component was clearly described or when phase II-related adherence outcomes could be extracted. Studies primarily focusing on long-term maintenance rehabilitation, general physical activity maintenance after completion of phase II CR, or phase III CR were excluded if phase II-specific intervention components or adherence outcomes could not be distinguished.

The exclusion criteria were as follows: (1) duplicate publications; (2) studies for which the full text was unavailable; (3) outdated guidelines that had been replaced by updated versions; and (4) guideline interpretation documents.

### 2.5 Study selection

Two reviewers (GH and CY), both trained in evidence-based medicine, independently performed the literature search according to the predefined strategy. After the search results were imported into EndNote, duplicate records were identified and removed using the software's deduplication function. Study selection was conducted in two stages.

In the first stage, titles, abstracts, and keywords were independently screened by the two reviewers against the predefined inclusion criteria, such as study population and intervention characteristics, to determine whether full-text review was required. Any disagreements regarding eligibility were resolved through discussion.

**Figure 1:**
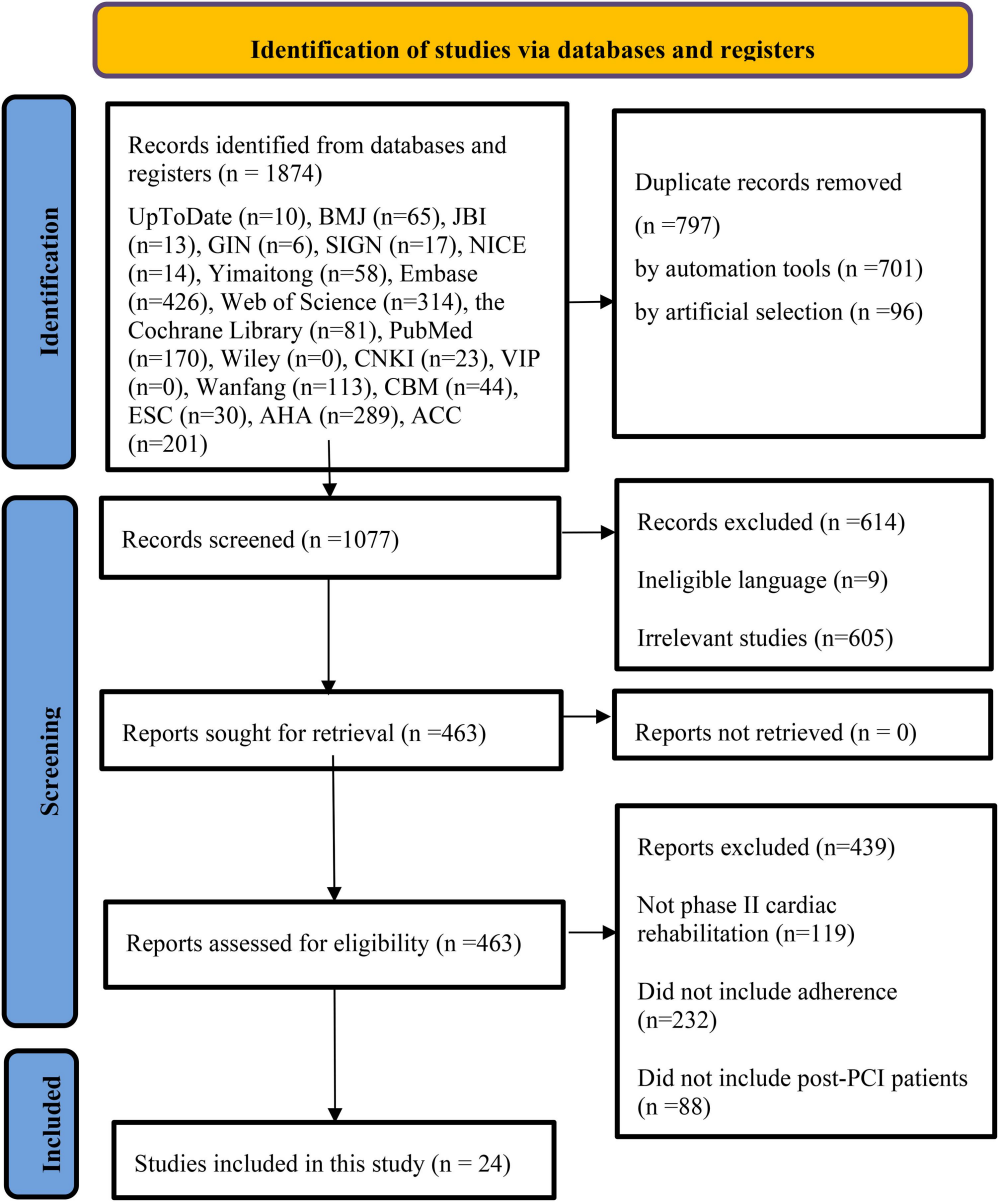
PRISMA flow diagram of literature search and selection process. JBI: the Joanna Briggs Institute; CMACPG: the Canadian Medical Association Clinical Practice Guidelines; GIN: the Guidelines International Network; SIGN: the Scottish Intercollegiate Guidelines Network; NICE: the National Institute for Health and Care Excellence; CNKI: China National Knowledge Infrastructure; CBM: Chinese Biology Medicine; ESC: the European Society of Cardiology; AHA: American Heart Association; ACC: American College of Cardiology.

In the second stage, full-text articles of potentially relevant studies were independently assessed by GH and CY in accordance with the predefined eligibility criteria and methodological standards. Final inclusion was determined through discussion. In cases of persistent disagreement, a third reviewer was consulted, and decisions were made based on the principles of prioritizing high-quality and recently published evidence. The detailed study selection process, including reasons for exclusion at each stage, is presented in Figure 1.

### 2.6 Literature quality assessment

Appropriate critical appraisal tools were selected according to the type of included evidence. Two reviewers (GH and CY) independently assessed the methodological quality of the included studies, and disagreements were resolved through discussion with a third reviewer.

Clinical guidelines were appraised using the 2017 updated Appraisal of Guidelines for Research and Evaluation II (AGREE II) instrument (27), which covers six domains: scope and purpose, stakeholder involvement, rigor of development, clarity of presentation, applicability, and editorial independence. Systematic reviews were evaluated using the JBI Critical Appraisal Checklist for Systematic Reviews and Research Syntheses (28) which consists of 11 items and assesses methodological quality in terms of the review question, inclusion criteria, search strategy, critical appraisal of included studies, data extraction, methods used to combine studies, publication bias, and whether the conclusions and recommendations are supported by the reported data. Each item was rated as “Yes,” “No,” “Unclear,” or “Not Applicable.” Randomized controlled trials (RCTs) were appraised using the Cochrane Risk of Bias 2.0 (ROB 2.0) tool (29), which evaluates the randomization process, deviations from intended interventions, missing outcome data, outcome measurement, and selection of the reported result. Expert consensus statements were appraised using the JBI Critical Appraisal Checklist for Text and Opinion Papers (30), which consists of six items and evaluates whether the source of opinion is clearly identified, whether the authors have standing in the relevant field, whether the interests of the target population are central to the opinion, whether the position is supported by analytical reasoning and logical argument, whether the existing literature is appropriately referenced, and whether any inconsistency with previous literature is addressed. Each item was likewise rated as “Yes,” “No,” “Unclear,” or “Not Applicable.” Quasi-experimental studies were appraised using the JBI Critical Appraisal Checklist for Quasi-Experimental Studies, which primarily evaluates causal clarity, comparability of participants, consistency of intervention delivery, outcome measurement, and completeness of follow-up (31).

For UpToDate clinical decisions or JBI evidence summaries, given the absence of dedicated appraisal tools for these resources, key original supporting studies were retrospectively traced, classified according to the research question, and appraised using the corresponding JBI tools (32).

### 2.7 Evidence extraction and synthesis

GH and CY independently extracted and synthesized the evidence according to the following principles:

1. practice-relevant evidence was prioritized while maintaining fidelity to the original text, accurately citing sources, and avoiding over-interpretation;
2. when overlapping evidence was identified, the most concise and representative statements were retained;
3. congruent or complementary evidence was integrated into unified evidence statements; and
4. when conflicting evidence was encountered, priority was given to evidence of higher quality and more recent publication.

Evidence was graded using the JBI Centre for Evidence-Based Medicine 2014 evidence grading system (Level 1 = highest, Level 5 = lowest) (33). Recommendation strength was subsequently determined in collaboration with three clinical specialists, one methodologist, and two nursing postgraduates using the JBI FAME framework (Feasibility, Appropriateness, Meaningfulness, and Effectiveness). Factors considered included intervention-related risks, cost–benefit balance, patient values, and willingness to participate. Recommendations were classified as Grade A (strong recommendation) or Grade B (weak recommendation) (33).

## 3 Results

### 3.1 Search results

Initially, 1874 records were identified through the systematic search, and after eliminating duplicates, 1,077 records were retained for screening. During the subsequent review and screening process, 24 articles met the inclusion criteria for this best evidence summary. Additionally, no relevant studies were found in the references of the included studies. Among these 24 studies, there were 1 clinical decision support resource (34), 6 guidelines (15, 24–26, 35), 6 expert consensus statements (23, 36–40), 6 systematic reviews (21, 22, 41–45), 4 RCTs (46–49) and 1 quasi-experimental study (50). The included studies were from the United States (*n* = 7) (34, 37, 39–41, 46), China (*n* = 6) (21, 23, 24, 38, 49, 50), Korea (*n* = 1) (15), Japan (*n* = 1) (35), Spain (*n* = 1) (44), Singapore (*n* = 1) (22), UK (*n* = 3) (25, 26, 47), New Zealand (*n* = 1) (43), Canada (*n* = 1) (45), Australia (*n* = 1) (48), and Europe (*n* = 2) (36, 51). Figure 1 shows the literature search process, and the characteristics of the included studies are summarized in Table 1.

**Table 1:** General information of the included literature.

Included literature	Source	Type of literature	Topic	Year of publication	Country/region
Wenger et al. (2025) (34)	UpToDate	Clinical decision	Cardiac rehabilitation in older adults	2025	USA
ESC (2024) (51)	ESC	Guideline	2024 ESC Guidelines for the management of chronic coronary syndromes	2024	Europe
Makita et al.(2022) (35)	PubMed	Guideline	JCS/JACR 2021 Guideline on Rehabilitation in Patients With Cardiovascular Disease	2022	Japan
Kim et al.(2019) (15)	PubMed	Guideline	Recommendations for Cardiac Rehabilitation and Secondary Prevention after Acute Coronary Syndrome	2019	Korea
Hu et al.(2018) (24)	CNKI	Guideline	Guidelines for cardiovascular rehabilitation and secondary prevention in China 2018 (simplified edition)	2018	China
SIGN (2017) (26)	SIGN	Guideline	Cardiac rehabilitation	2017	UK
NICE (2020) (25)	NICE	Guideline	Acute coronary syndromes	2020	UK
Coutinho et al.(2025) (37)	AHA	Expert Consensus	Cardiac Rehabilitation in Women: A Scientific Statement From the American Heart Association	2025	USA
Golbus et al.(2023) (39)	AHA	Expert Consensus	Digital Technologies in Cardiac Rehabilitation: A Science Advisory From the American Heart Association	2023	USA
Chinese expert consensus(2022) (23)	Yimaitong	Expert Consensus	Expert consensus on integrated traditional Chinese and Western medicine cardiac rehabilitation after percutaneous coronary intervention	2022	China
Experts Consensus Group(2022) (38)	Yimaitong	Expert Consensus	Home-based cardiac rehabilitation (HBCR) for patients with cardiovascular diseases in China	2022	China
EAPC (2021) (36)	ESC	Expert Consensus	Secondary prevention through comprehensive cardiovascular rehabilitation: From knowledge to implementation. 2020 update.	2021	Europe
Thomas et al.(2019) (40)	AHA	Expert Consensus	Home-Based Cardiac Rehabilitation	2019	USA
Xia et al.(2024) (21)	PubMed	Systematic review	Comparative effectiveness of different interventions on adherence to exercise-based CR among patients after percutaneous coronary intervention: a network meta-analysis of randomized controlled trials	2024	China
Ramachandran et al.(2022) (22)	ESC	Systematic review	Effectiveness of home-based cardiac telerehabilitation as an alternative to Phase 2 cardiac rehabilitation of coronary heart disease	2022	Singapore
de Araújo Pio et al.(2019) (45)	PubMed	Systematic review	Interventions to Promote Patient Utilization of Cardiac Rehabilitation: Cochrane Systematic Review and Meta-Analysis	2019	Canada
Edwards et al.(2019) (41)	PubMed	Systematic review	Depression Is Associated With Reduced Outpatient Cardiac Rehabilitation Completion Rates. A systematic literature review and meta-analysis	2019	USA
Rawstorn et al.(2016) (43)	PubMed	Systematic review	Telehealth exercise-based cardiac rehabilitation: a systematic review and meta-analysis	2016	New Zealand
Ruano-Ravina et al.(2016) (44)	Web of Science	Systematic review	Participation and adherence to cardiac rehabilitation programs. A systematic review.	2016	Spain
Zhang et al.(2025) (49)	PubMed	Randomized controlled trial	Effectiveness of Smartwatch Device on Adherence to Home-Based Cardiac Rehabilitation in Patients With Coronary Heart Disease: Randomized Controlled Trial	2025	China
Taylor et al.(2020) (48)	PubMed	Randomized controlled trial	Short-term and Long-term Feasibility, Safety, and Efficacy of High-Intensity Interval Training in Cardiac Rehabilitation The FITR Heart Study Randomized Clinical Trial	2020	Australia
Gaalema et al.(2019) (46)	PubMed	Randomized controlled trial	Financial Incentives to Increase Cardiac Rehabilitation Participation among Low-Socioeconomic Status Patients: A Randomized Clinical Trial	2019	USA
Sniehotta et al.(2006) (47)	PubMed	Randomized controlled trial	Action plans and coping plans for physical exercise: A longitudinal intervention study in cardiac rehabilitation	2006	UK
Jin et al.(2024) (50)	PubMed	Quasi-experimental study	A study on the intervention effect of a case management model that breaks through spatiotemporal characteristics in home-based phase II exercise rehabilitation post PCI	2024	China

### 3.2 Quality evaluation results of the included literature

#### 3.2.1 Quality evaluation result of clinical decisions

One clinical decision from the UpToDate platform was initially included. Two original supporting references were traced, including one expert consensus statement (52) and one systematic review (53). Quality appraisal showed that the expert consensus statement was of acceptable quality and the systematic review was of high methodological quality. Accordingly, the relevant evidence derived from this UpToDate clinical decision was retained.

#### 3.2.2 Quality assessment results of guidelines

This study included 6 guidelines. The standardized domain scores and overall quality assessment results for each guideline are presented in Table 2.

**Table 2:** Methodological quality evaluation results of the guidelines.

Guideline	Standardized scores in various domains (%)						≥60%	≤30%	Quality evaluation
	①	②	③	④	⑤	⑥			
ESC (2024) (51)	93.3	86.7	83.3	96.7	66.7	91.7	6	0	A
Makita et al. (2022) (35)	93.3	81.7	76.7	93.3	71.7	86.7	6	0	A
Kim et al. (2019) (15)	90	80	78.3	92	73.3	86.7	6	0	A
Hu et al. (2018) (24)	86.7	73.3	71.7	91.7	66.7	83.3	6	0	A
SIGN (2017) (26)	93.3	85	81.7	94	80	90	6	0	A
NICE (2020) (25)	93.3	86.7	88.3	96.7	83.3	86.7	6	0	A

① Scope and purpose；② Stakeholder involvement；③ Rigor of development；④ Clarity of presentation；⑤ Applicability；⑥ Editorial independence.

#### 3.2.3 Quality assessment results of expert consensus statements

This study included 6 expert consensus statements. The quality assessment results are presented in Table 3.

**Table 3:** Methodological quality evaluation of expert consensus statements.

Items	Coutinho et al.(2025) (37)	Golbus et al.(2023) (39)	Chinese expert consensus (2022) (23)	Experts Consensus Group(2022) (38)	EAPC (2021) (36)	Thomas et al.(2019) (40)
①	Yes	Yes	Yes	Yes	Yes	Yes
②	Yes	Yes	Yes	Yes	Yes	Yes
③	Yes	Yes	Yes	Yes	Yes	Yes
④	Yes	Yes	Yes	Yes	Yes	Yes
⑤	Yes	Yes	Yes	Yes	Yes	Yes
⑥	Yes	Yes	Yes	Yes	Yes	Yes

① Is the source of the opinion clearly identified? ② Does the source of opinion have standing in the field of expertise? ③ Are the interests of the relevant population the central focus of the opinion? ④ Is the stated position the result of an analytical process, and is there logic in the opinion expressed? ⑤ Is there reference to the extant literature? ⑥ Is any incongruence with the literature/sources logically defended?

#### 3.2.4 Quality assessment results of systematic reviews

This study included 6 systematic reviews and meta-analyses. Overall, the methodological quality of the included studies was relatively high, with most items rated as “Yes.” However, some methodological limitations remained, particularly in the assessment of publication bias. Detailed quality assessment results are presented in Table 4.

**Table 4:** Methodological quality evaluation of systematic reviews.

Item	Edwards et al.(2019) (41)	de Araújo Pio et al.(2019) (45)	Rawstorn et al.(2016) (43)	Ramachandran et al. (2022) (22)	Xia et al. (2024) (21)	Ruano-Ravina et al.(2016) (44)
①	Yes	Yes	Yes	Yes	Yes	Yes
②	Yes	Yes	Yes	Yes	Yes	Yes
③	No	Yes	Yes	Yes	Yes	Yes
④	No	Yes	Yes	Yes	Yes	Yes
⑤	Yes	Yes	Yes	Yes	Yes	Unclear
⑥	Yes	Yes	Yes	Yes	Yes	Unclear
⑦	Yes	Yes	No	Yes	Yes	Unclear
⑧	Yes	Yes	Yes	Yes	Yes	Yes
⑨	No	Yes	No	No	Yes	No
⑩	Yes	Yes	Yes	Yes	Yes	Yes
⑪	Yes	Yes	Yes	Yes	Yes	Yes

① Is the review question clearly and explicitly stated? ② Were the inclusion criteria appropriate for the review question? ③ Was the search strategy appropriate? ④ Were the sources and resources used to search for studies adequate? ⑤ Were the criteria for appraising studies appropriate? ⑥ Was critical appraisal conducted by two or more reviewers independently? ⑦ Were there methods to minimize errors in data extraction? ⑧ Were the methods used to combine studies appropriate? ⑨ Was the likelihood of publication bias assessed? ⑩ Were recommendations for policy and/or practice supported by the reported data? ⑪ Were the specific directives for new research appropriate?

#### 3.2.5 Quality assessment results of randomized controlled trials

This study included 4 randomized controlled trials. The overall risk-of-bias judgments and domain-specific assessment results for each trial are presented in Table 5.

**Table 5:** Methodological quality evaluation of randomized controlled trials.

Author	Randomization process	Deviations from the intended interventions	Missing outcome data	Measurement of the outcome	Selection of the reported result	Overall bias
Zhang et al.(2025) (49)	Some concerns	Low risk	Some concerns	Some concerns	Some concerns	Some concerns
Taylor et al.(2020) (48)	Low risk	Low risk	Some concerns	Some concerns	Low risk	Some concerns
Gaalema et al.(2019) (46)	Some concerns	Low risk	Low risk	Low risk	Low risk	Some concerns
Sniehotta et al.(2006) (47)	Some concerns	Some concerns	Some concerns	Some concerns	Some concerns	Some concerns

#### 3.2.6 Quality assessment results of quasi-experimental studies

This study included 1 quasi-experimental study. Overall, the methodological quality of the included study was high, with all appraisal items rated as “Yes.” Detailed quality assessment results are presented in Table 6**.**

**Table 6:** Methodological quality evaluation of the quasi-experimental study.

Item	Bias related to temporal precedence	Bias related to selection and allocation	Bias related to confounding factors	Bias related to administration of intervention/exposure	Bias related to assessment, detection and measurement of the outcome			Bias related to participant retention	Statistical Conclusion Validity	Overall quality
	①	②	③	④	⑤	⑥	⑦	⑧	⑨	
Jin et al.(2024) (50)	Yes	Yes	Yes	Yes	Yes	Yes	Yes	Yes	Yes	High

①Is it clear in the study what is the “cause” and what is the “effect” (i.e., there is no confusion about which variable comes first)? ② Was there a control group? ③ Were participants included in any comparisons similar? ④ Were the participants included in any comparisons receiving similar treatment/care, other than the exposure or intervention of interest? ⑤ Were there multiple measurements of the outcome, both pre and post the intervention/exposure? ⑥ Were the outcomes of participants included in any comparisons measured in the same way? ⑦ Were outcomes measured in a reliable way? ⑧ Was follow-up complete and if not, were differences between groups in terms of their follow-up adequately described and analyzed? ⑨ Was appropriate statistical analysis used?

### 3.3 Summary and description of evidence

**Figure 2:**
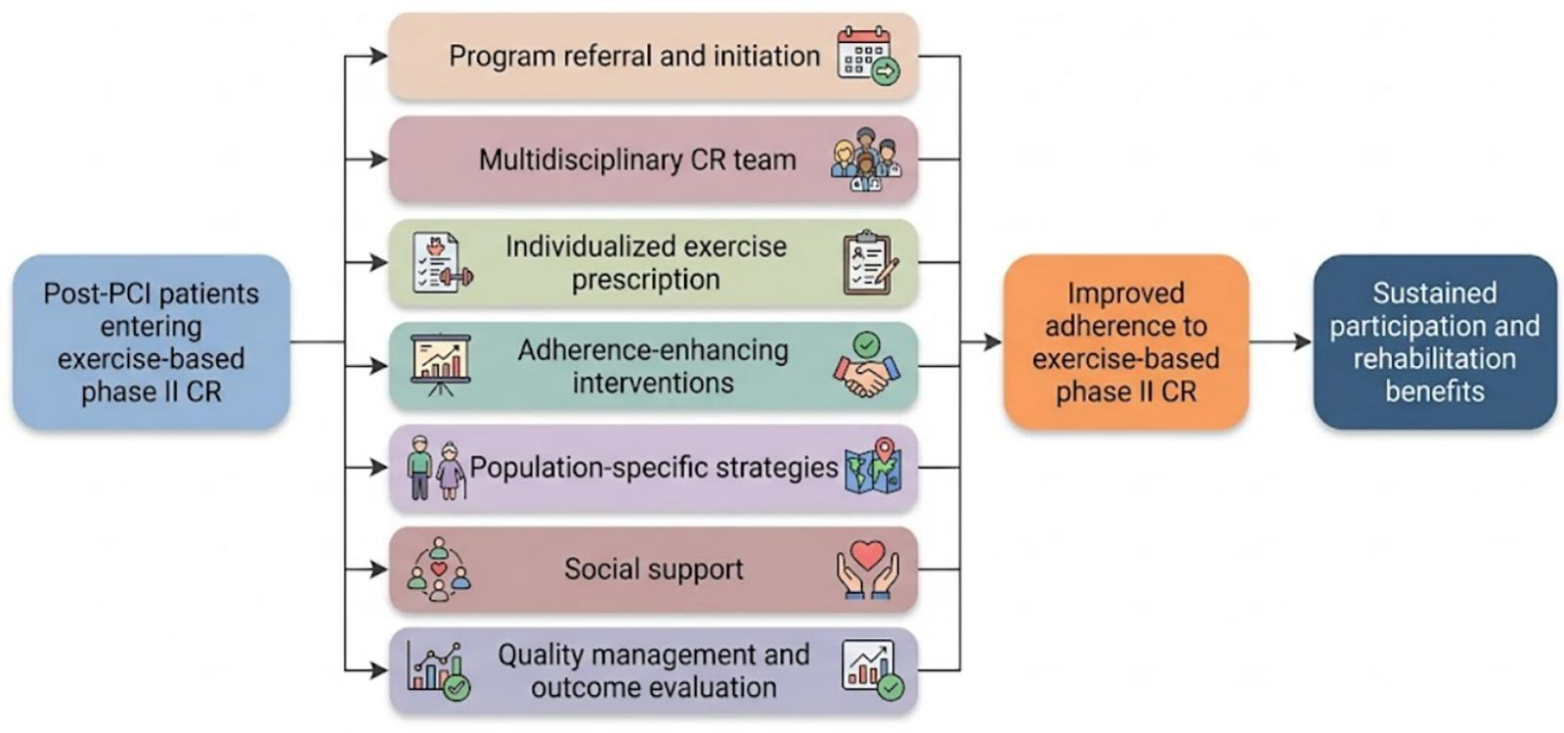
Conceptual framework of evidence-based strategies for improving adherence to exercise-based phase II cardiac rehabilitation after PCI.

Based on the principles of evidence synthesis and in combination with clinical practice, a total of 32 pieces of evidence related to improving adherence to exercise-based phase II cardiac rehabilitation in patients after PCI were summarized. These evidence findings were grouped into 7 dimensions: program referral and initiation, the multidisciplinary cardiac rehabilitation team, individualized exercise prescription, adherence-enhancing interventions, intervention strategies for special populations, social support, and quality management and outcome evaluation, as shown in Table 7. To further illustrate the overall structure and practical logic of the synthesized evidence, a conceptual framework was developed to provide an overview of how these seven evidence dimensions may collectively contribute to improving adherence to exercise-based phase II CR after PCI and supporting subsequent rehabilitation benefits (Figure 2). Where adherence-related quantitative findings were directly reported in the included evidence, they are described in the corresponding sections of the Discussion to support interpretation of the evidence statements.

**Table 7:** Summary of best evidence for improving adherence to exercise-based phase II cardiac rehabilitation after percutaneous coronary intervention.

Category	ID	Content of evidence	Level	Recommendation level
Program Referral and Initiation	1	Eligible patients after PCI should be referred to an outpatient cardiac rehabilitation (CR) program before hospital discharge. (15, 24, 25)	5b	A
	2	Automatic referral and liaison systems should be considered to increase CR referral and uptake. (15, 34, 45)	5b	A
	3	Patients referred to a CR program should receive an early individualized assessment to facilitate timely contact with the CR team, identify rehabilitation needs and barriers, and support subsequent uptake and ongoing attendance. (26)	5b	A
	4	Before initiating phase II exercise-based CR, a comprehensive baseline assessment is recommended to support safety screening, risk stratification, and individualized exercise prescription. This assessment should include evaluation of exercise capacity and functional status, relevant comorbidities and musculoskeletal or functional limitations, lifestyle-related risk factors, and psychosocial status. (15, 24, 35, 36, 51)	5b	A
	5	For clinically stable patients after PCI, phase II exercise-based CR should be initiated as early as feasible after discharge, with the initial CR session preferably starting within 10 days after discharge. (15, 24, 25, 36)	5b	A
Multidisciplinary Cardiac Rehabilitation Team	6	It is recommended to establish a multidisciplinary team that includes cardiologists, CR nurses, rehabilitation specialists with expertise in CR, and psychologists or other mental health professionals. (15, 35, 36)	5b	A
	7	CR physicians are responsible for systematic patient assessment and formulation of the rehabilitation program; CR nurses are responsible for establishing patient records, documenting data, monitoring progress, and providing guidance; rehabilitation specialists play a leading role in delivering exercise training; and psychologists or counselors are responsible for the assessment and management of patients’ psychological problems. (15, 23, 24, 35, 36)	5b	A
Individualized Exercise Prescription	8	Individualized exercise prescriptions should be developed based on patients’ clinical status, exercise capacity and functional status, relevant comorbidities, and personal preferences. (15, 23, 35, 51)	5b	A
	9	Exercise prescriptions should follow the FITT-VP principle, including frequency, intensity, time, type, volume, and progression (15, 24, 35, 36)	5b	A
	10	Exercise training programs should adopt a progressive training strategy, with gradual increases in exercise duration, intensity, or frequency based on patient tolerance (24, 34–36).	5b	A
	11	Both high-intensity interval training (HIIT) and moderate-intensity continuous training (MICT) can be incorporated into CR programs (48), with exercise intensity individualized according to clinical status, exercise capacity, and safety considerations (35, 36).	1c	A
	12	Home-based cardiac rehabilitation supported by mobile health is recommended as an effective strategy for improving adherence to phase II exercise-based CR after PCI (21).	1a	A
	13	Home-based cardiac rehabilitation is generally appropriate for selected, clinically stable patients at low cardiovascular risk and for some carefully selected patients at moderate cardiovascular risk (22, 36, 38, 40).	5b	A
	14	For clinically stable patients at intermediate or high cardiovascular risk, a hybrid model combining supervised outpatient CR with home-based exercise training is recommended (38, 40).	5b	A
	15	Exercise plans are recommended to incorporate “if-then” coping plans for anticipated barriers (47).	1b	A
Adherence Interventions	16	Standardized indicators, such as attendance, completion of prescribed sessions, and adherence to FITT-VP exercise parameters, are recommended for assessing exercise adherence in CR. (21, 44, 45)	1a	A
	17	Structured patient education covering exercise prescription, self-monitoring, and cardiovascular risk factor management should be provided. (15, 25, 35, 36, 40, 51)	5b	A
	18	Healthcare professionals involved in CR should receive training in motivational interviewing, patient-centered communication, and shared decision-making. (36, 51)	5b	A
	19	Motivational interviewing, shared decision-making, and effective patient-provider communication should be incorporated into CR practice to support behavior change and improve exercise adherence. (26, 36, 45, 51)	1b	A
	20	Digital support tools, such as wearable devices and mobile health applications, are recommended to facilitate data sharing, remote monitoring, and real-time feedback, thereby enhancing patient engagement and adherence to exercise programs. (21, 39, 43, 49, 51)	1a	A
	21	Patients who do not start or do not continue to attend a CR program should be actively re-contacted using reminder strategies, such as motivational letters, telephone calls, prearranged visits from CR team members, or combined approaches, to promote program initiation and continued attendance. (25)	5b	B
	22	Case-management-based continuous care may be considered as an adjunct to home-based phase II CR after PCI to support exercise adherence and regular follow-up. (50)	2c	B
Population-Specific Strategies	23	Adherence support strategies should address the needs of specific populations, including women, older adults, patients with depression, and patients with low socioeconomic status. (34, 37, 41, 46, 51)	5b	A
	24	Women should be assessed for psychosocial and gender-related barriers to participation in CR, and supportive approaches tailored to women's needs may be considered to facilitate engagement. (37)	5b	B
	25	Exercise prescriptions for women should be individualized according to physiological characteristics, comorbidities, functional status, and personal preferences. (37)	5b	B
	26	Frailty and fall risk should be assessed in older adults to identify reduced physiological reserve, multimorbidity, and rehabilitation-related safety concerns. (34)	5b	A
	27	Balance and flexibility training may be incorporated into individualized exercise programs for older adults according to functional status and safety needs. (34)	5b	B
	28	Patients with depression, anxiety, or other psychosocial problems should be provided with, or referred for, appropriate psychological support as an integral component of exercise-based CR. (15, 26, 36)	5b	A
	29	Financial incentive interventions may be considered for patients with low socioeconomic status to reduce barriers to participation in CR and improve adherence. (45, 46)	1c	B
Social Support	30	Family involvement may be encouraged in home-based CR, and targeted education may be provided to support home exercise and self-management. (23, 38)	5b	B
	31	Support from peer volunteers or peer advisors with previous cardiac event and CR experience may be considered as a potentially supportive approach to promote CR utilisation. (45)	1b	B
Quality Management and Outcome Evaluation	32	A CR database or registry should be established to record exercise data, clinical indicators, adverse events, and key outcomes such as exercise capacity and adherence, with regular evaluation of these indicators. (24, 35, 36)	5b	A

## 4 Discussion

### 4.1 Program referral and initiation

Evidence 1–5 summarized the key strategies for referral to and initiation of exercise-based cardiac rehabilitation programs, including referral to cardiac rehabilitation before discharge, automated referral systems, early individualized assessment, comprehensive baseline assessment and early program initiation. Implementing an automated referral system at the time of discharge can effectively reduce missed referrals and significantly improve enrollment in cardiac rehabilitation programs. Grace et al. (54) found that, compared with the traditional physician-directed referral approach, a model combining automatic referral with healthcare provider endorsement significantly increased patient participation in cardiac rehabilitation. After referral, patients should receive an early individualized assessment to facilitate timely contact with the CR team and identify clinical risk, exercise capacity, psychological status, rehabilitation needs, and practical barriers to participation (15, 24, 26, 35, 36, 51). This assessment can inform individualized rehabilitation planning and help develop exercise-based rehabilitation plans that are more acceptable and practicable for patients (5). Following comprehensive baseline assessment and individualized rehabilitation planning, clinically stable patients should initiate phase II exercise-based CR as early as feasible after discharge, with the initial CR session preferably starting within 10 days after discharge. Studies have shown that earlier participation is more conducive to establishing exercise habits, preventing behavioral inertia associated with prolonged postoperative recovery, and facilitating timely engagement in rehabilitation (55). However, in some regions, cardiac rehabilitation resources remain insufficient. Effective coordination between inpatient care teams and rehabilitation teams has not yet been established, and standardized referral pathways and integrated rehabilitation network systems are still lacking, which highlights the need for institution-level coordination and system-level support (56). In these regions, healthcare teams should make full use of existing resources, strengthen collaboration between inpatient medical teams and post-discharge rehabilitation teams, and establish standardized referral processes and systems.

### 4.2 Multidisciplinary cardiac rehabilitation team

Evidence 6–7 support the delivery of exercise-based phase II cardiac rehabilitation after PCI through a multidisciplinary team and emphasize the importance of clearly defined professional roles (15, 23, 24, 35, 36). The value of the multidisciplinary team model lies in its ability to integrate key components of rehabilitation, including medical assessment, exercise training, monitoring, patient education, follow-up, and psychosocial care, thereby supporting coordinated and comprehensive rehabilitation management (15, 23, 35, 36). However, translating this model into actual rehabilitation benefits depends on effective collaboration and adequate implementation conditions. In practice, insufficient human resources, time constraints, inadequate communication among team members, and unclear role boundaries may limit team effectiveness (24, 35, 36, 57). Therefore, team members should be allocated according to available resources, their responsibilities should be clearly defined, and standardized communication and referral pathways should be established (24, 35, 36). Regular case discussions or team meetings may further strengthen collaboration, where feasible, a dedicated coordinator may help improve team functioning and continuity of rehabilitation management.

### 4.3 Individualized exercise prescription

Evidence 8–15 indicate that individualized exercise prescription represents a core strategy for improving adherence to exercise-based phase II cardiac rehabilitation after PCI. In clinical practice, exercise programs that are more closely tailored to patients' clinical status, exercise capacity, comorbidities, functional limitations, and personal preferences are more likely to be accepted and maintained over time (15, 23, 35, 51). Current guidelines recommend that exercise prescription should follow the FITT-VP principle (15, 24, 35, 36), which provides a structured framework for determining exercise frequency, intensity, time, type, volume, and progression. Within this framework, progressive training is particularly important. Gradual adjustment of exercise duration, intensity, or frequency according to patient tolerance may help patients adapt progressively to rehabilitation demands while reducing excessive fatigue and potential cardiovascular risk (24, 34–36). Regarding exercise intensity strategies, both high-intensity interval training (HIIT) and moderate-intensity continuous training (MICT) are feasible options within cardiac rehabilitation. Existing systematic reviews and meta-analyses suggest that HIIT may be superior to MICT in improving peak oxygen uptake (VO2peak), whereas no significant difference has been observed in health-related quality of life; moreover, when only isocaloric protocols are considered, the difference in VO2peak improvement is also not significant (58). Therefore, exercise intensity should be individualized according to clinical status, exercise capacity, patient tolerance, and safety considerations (35, 36).

Beyond exercise content, the rehabilitation delivery pathway also influences rehabilitation outcomes and adherence. Existing evidence suggests that mobile health-supported home-based cardiac rehabilitation can improve adherence to phase II exercise rehabilitation after PCI, but this model is best suited to low-risk, clinically stable patients and some carefully selected moderate-risk patients (22, 36, 38, 40). Xia et al. conducted the network meta-analysis of 30 randomized controlled trials involving 4,267 post-PCI patients and found that home-based CR combined with mobile health intervention ranked highest for improving adherence to exercise-based phase II CR, with the highest surface under the cumulative ranking curve (SUCRA) value of 83.8%, followed by hospital-based CR combined with mobile health intervention, with a SUCRA value of 79.9% (21). For patients at moderate or higher cardiovascular risk, a hybrid model combining supervised outpatient training with home-based exercise is generally more appropriate for balancing adherence and safety (40, 59).

Furthermore, individualized exercise prescription should not be limited to the specification of exercise parameters and delivery modes alone. Anticipated barriers during rehabilitation, such as time conflicts, physical discomfort, and environmental changes, can also interrupt sustained participation in exercise. Therefore, incorporating “if–then” coping plans into rehabilitation programs can help patients prepare specific responses to these common barriers, thereby strengthening self-regulation and maintaining continuity of exercise behavior (47).

However, some limitations remain. Although current evidence supports selecting home-based or hybrid pathways according to risk stratification, more refined evidence is still needed regarding the optimal level of supervision, timing of transition, and subsequent management for patients at different risk levels. Likewise, although “if–then” coping plans show practical promise, their optimal implementation and long-term effects in exercise-based phase II cardiac rehabilitation after PCI remain to be established.

### 4.4 Adherence-enhancing interventions

Evidence 16–22 summarize the main strategies for improving adherence to phase II exercise-based cardiac rehabilitation after PCI, including adherence assessment, structured education, communication-based behavioural support, digital support, active reminder and re-contact strategies and follow-up management. First, adherence can be assessed using standardized indicators such as attendance, completion of prescribed exercise, and attainment of FITT-VP parameters. These indicators may help healthcare professionals more objectively evaluate patients' actual exercise performance, identify individuals with insufficient adherence, and provide a basis for subsequent individualized intervention (21). Second, structured education on exercise prescription, self-monitoring, and cardiovascular risk factor management remains a fundamental component of adherence support, as it can enhance patients' understanding of the necessity of exercise rehabilitation and the requirements of self-management, thereby improving exercise adherence (21, 24, 45).

However, educational support alone may still be insufficient for patients who face motivational or practical barriers to participation. Patient-centred communication and shared decision-making should therefore be incorporated into CR practice as important behavioural support strategies. Through open communication, discussion of rehabilitation goals, and negotiation of feasible plans, these approaches may help healthcare professionals better identify patients' key concerns, practical needs, and perceived barriers, and develop rehabilitation plans that are more understandable, acceptable, and actionable for patients (21, 45). Motivational interviewing may be applied as a supportive counselling strategy, particularly for patients who experience ambivalence, low confidence, or other barriers to participation (26, 36, 51). Nevertheless, the standardized implementation of these strategies depends not only on institutional support and service-process arrangements, but also on whether healthcare professionals possess the necessary communication and behavioural-support competencies. Strengthening training in motivational interviewing, effective communication, and behavioural support is therefore an important prerequisite for the effective implementation of such interventions (51).

With the development of digital health, wearable devices and mobile health applications can strengthen post-discharge behavioural tracking and adherence support through automatic exercise data synchronization, remote monitoring, and real-time feedback. Existing evidence suggests that digitally supported interventions, particularly those incorporating mobile health functions, may improve adherence to exercise-based cardiac rehabilitation after PCI by strengthening behavioural tracking and timely feedback during the post-discharge period (21, 39, 40). Zhang et al. found that, compared with standard home-based CR, smartwatch-facilitated home-based CR produced higher 3-month exercise adherence scores, as measured by the Home-Based Exercise Training Adherence Questionnaire (89.17 ± 9.29 vs. 72.50 ± 17.17, *P* = .003) (47). For patients who do not start or do not continue to attend CR, active reminder and re-contact strategies will also help promote program initiation and continued attendance. These strategies may include motivational letters, telephone calls, prearranged visits or contacts from CR team members, or combined approaches (25, 45). In clinical practice, these strategies may be implemented by identifying patients who miss the initial CR session or interrupt attendance, providing timely reminder contacts, and documenting patients' reasons for non-participation to guide subsequent individualized support. In addition, case-management-based continuous care may provide a more structured approach to post-discharge follow-up, particularly in home-based phase II CR after PCI. Through regular assessment, individualized guidance, exercise monitoring, and continuous communication, case management may help maintain rehabilitation continuity and support exercise adherence after discharge (50).

However, several limitations remain. First, definitions and measures of adherence vary substantially across studies. In addition to attendance, completion, and attainment of FITT parameters, questionnaires, self-reports, and platform-based records have also been used, which may reduce comparability across studies (21). Second, most mobile health- and remote support-based interventions have been evaluated in patients with relatively stable conditions, better baseline adherence, or a certain level of digital literacy. Their use in older adults, frail individuals, or populations with limited digital resources remains challenging in practice and may require simplified interface design, additional technical support, or alternative non-digital follow-up pathways; the effectiveness of such approaches still requires further validation (34, 39, 40). Third, although reminder and re-contact strategies are practical and low-risk, their effectiveness may depend on accurate identification of patients who do not start or discontinue CR and timely follow-up documentation. In addition, evidence on case-management-based continuous care remains limited and should be further confirmed in larger samples or randomized controlled trials.

### 4.5 Intervention strategies for specific populations

#### 4.5.1 Women

Existing studies show that participation in and adherence to cardiac rehabilitation are generally lower among women than among men. A meta-analysis of 14 studies involving 8,176 participants found that (60) women accounted for only 27.3% of cardiac rehabilitation participants, and their adherence was also lower, suggesting greater barriers to both enrollment and sustained participation. These disparities are associated with women's social role burdens, inadequate adaptation of rehabilitation environments, and insufficient attention to psychosocial support needs. For example, family caregiving responsibilities, work-related conflicts, and time pressures often limit women's regular participation (37, 61). Therefore, support for female patients should extend beyond general adjustments of exercise prescriptions and should first identify psychosocial and gender-related barriers to participation and sustained engagement. Exercise prescriptions should also be individualized according to women's physiological characteristics, comorbidities, functional status, and personal preferences to improve acceptability and sustainability (37). However, the barriers faced by women are not uniform across cultural and social contexts. Among women from ethnic minority groups in particular, cultural norms, religious beliefs, and gender role expectations may further shape rehabilitation participation, highlighting the need for greater cultural sensitivity and attention to individual differences in future intervention design (37, 61).

#### 4.5.2 Older adults

Among older adults, reduced exercise adherence is related not only to motivation, but also to diminished physiological reserve, frailty, multimorbidity, and increased fall risk. Older patients often present with decreased muscle strength, impaired balance, gait instability, and polypharmacy, all of which may increase exercise-related discomfort and fall risk (62). If these factors are not adequately recognized, patients may discontinue rehabilitation because of discomfort, fear of falling, or low self-efficacy. The AHA/AACVPR core components document (5) identifies frailty and fall risk as important assessment domains. Incorporating balance and flexibility training into individualized exercise programs has been shown to improve balance and reduce fall risk (34, 63). Such training may also enhance acceptance of and persistence with rehabilitation by improving physical stability and reducing concerns about falling (63, 64). However, the evidence base for cardiac rehabilitation in older adults remains limited. Existing studies show substantial heterogeneity in frailty assessment tools and study design, while randomized controlled trial evidence remains relatively scarce (65). In addition, although balance and coordination training shows promise for improving balance and reducing fall risk, its optimal implementation in phase II cardiac rehabilitation after PCI among older adults still requires further investigation.

#### 4.5.3 Patients with psychological problems

Psychological problems such as depression and anxiety are not uncommon among patients undergoing cardiac rehabilitation. A large study (66) found that the prevalence of moderate depression, anxiety, and stress symptoms was 18%, 28%, and 13%, respectively, and that patients with these symptoms were significantly less likely to continue cardiac rehabilitation than those with normal or mild symptoms. A systematic review (41) further showed that depression was associated with lower completion rates of outpatient cardiac rehabilitation. These findings highlight the importance of incorporating psychological screening into the comprehensive assessment before rehabilitation begins, so that affected patients can be identified early and provided with targeted psychological support or referred to mental health professionals when necessary. However, current evidence mainly supports the general principle that psychological support should be integrated into the rehabilitation pathway, whereas the optimal type, frequency, and long-term effects of support for different psychological conditions remain unclear (15, 36).

#### 4.5.4 Patients with low socioeconomic status

Patients with low socioeconomic status are another group requiring particular attention. Existing studies indicate that lower income, limited transportation access, and the financial burden associated with rehabilitation programs may reduce participation in and sustained adherence to cardiac rehabilitation (67, 68). Therefore, interventions for this population should focus on identifying and alleviating practical barriers to participation. A randomized clinical trial by Gaalema et al. in Medicaid-covered patients with low socioeconomic status showed that progressively increasing financial incentives significantly increased the number of completed cardiac rehabilitation sessions (22.4 vs. 14.7, *P* = 0.013) and nearly doubled program completion rates (55.4% vs. 29.2%, *P* = 0.002) (46). These findings suggest that financial incentives may help reduce participation barriers and improve program completion in selected settings. Nevertheless, the generalizability of this evidence should be interpreted cautiously, because it is derived mainly from a single randomized trial conducted in a Medicaid population within a specific healthcare system. Further research is needed to determine support strategies that are better adapted to different cultural and healthcare contexts.

### 4.6 Social support

Social support may support participation in exercise-based phase II cardiac rehabilitation after PCI, particularly during home-based rehabilitation or the continuation phase after supervised training. Current evidence suggests that family involvement may be encouraged in home-based cardiac rehabilitation, and that targeted education may help family members support home exercise and self-management. However, the way such support is delivered remains important (69, 70). Excessive protection or assuming rehabilitation tasks on behalf of patients may reduce patients' confidence and active participation in rehabilitation (23, 38).

Peer support may also be considered a potentially supportive strategy in exercise-based cardiac rehabilitation. Through shared experiences and mutual encouragement, it may help strengthen patients' confidence in rehabilitation participation. However, its independent effect on exercise adherence remains difficult to determine, because peer support is often embedded within broader multicomponent interventions rather than evaluated as a standalone strategy (45).

Overall, current evidence suggests that family and peer support may provide additional support for home exercise and self-management, but the evidence base remains limited. Evidence on family involvement is derived mainly from guidelines, consensus statements, and observational studies, whereas high quality intervention studies directly examining its independent effect on exercise adherence remain scarce. Similarly, substantial variation in the forms, duration, and implementation of peer support interventions limits comparability across studies. Future research should further clarify how different forms of social support influence exercise adherence (23, 38, 45).

### 4.7 Quality management and outcome evaluation

The implementation of rehabilitation quality management and outcome evaluation is an important foundation for ensuring the standardized delivery and continuous improvement of exercise-based phase II cardiac rehabilitation after PCI (24, 35, 36). By establishing databases and conducting structured documentation, dynamic assessment, and continuous follow-up with feedback, a more structured rehabilitation management process can be supported, thereby providing a basis for optimizing exercise prescriptions, monitoring adherence, and ensuring safety management. Given that this type of evidence is derived mainly from guidelines, its recommendations primarily reflect basic requirements at the management level. Therefore, in practice, localized implementation remains necessary according to the institution's information infrastructure, follow-up capacity, and resource allocation.

## 5 Limitations

This study systematically summarized the available evidence on strategies to improve adherence to exercise-based phase II cardiac rehabilitation among post-PCI patients. However, several limitations should be acknowledged. First, only literature published in Chinese and English was included, which may have resulted in the omission of relevant high-quality studies published in other languages. Future evidence updates may therefore benefit from broader language inclusion and the incorporation of newly published research. Second, the included evidence was derived from multiple countries, and substantial heterogeneity existed in intervention measures and implementation contexts. This heterogeneity may limit the formulation of fully standardized recommendations. Therefore, the interpretation and application of these findings should take the specific clinical context into account. Third, although this review primarily focused on post-PCI patients undergoing phase II exercise-based cardiac rehabilitation, some of the included evidence was informed by broader cardiac rehabilitation guidelines and expert consensus statements for coronary heart disease populations that included patients after PCI. This should be taken into account when interpreting the specificity of the findings. Fourth, although multiple high-quality evidence sources were included, potential bias cannot be completely excluded. Expert consensus statements and clinical guidelines may be influenced by the perspectives and experience of the contributing experts, thereby introducing a degree of subjectivity. In addition, some evidence was derived from non-randomized studies, which may be subject to confounding and may limit the generalizability of the findings. Future research should place greater emphasis on well-designed randomized controlled trials and other high-quality studies to further validate the effectiveness and applicability of the proposed strategies.

## 6 Conclusion

A total of 32 evidence statements on strategies to improve adherence to exercise-based phase II cardiac rehabilitation in post-PCI patients were summarized in this study, providing an evidence-informed basis for clinical practice. However, evidence in some areas remains limited and heterogeneous. Therefore, healthcare professionals should interpret and apply these findings in light of the specific clinical context, available resources, and patient preferences. Future research should focus on identifying effective, targeted, and context-sensitive strategies that are acceptable to patients and can improve adherence to exercise-based phase II cardiac rehabilitation among post-PCI patients.

## Data Availability

The original contributions presented in the study are included in the article/Supplementary Material, further inquiries can be directed to the corresponding author/s.
