# Supplementary material for: Strategies to improve adherence to exercise-based phase II cardiac rehabilitation after percutaneous coronary intervention: a best evidence summary
Source: Front Cardiovasc Med. 2026 Jul 15;13:1868542. doi: 10.3389/fcvm.2026.1868542 (PMC13415501; doi:10.3389/fcvm.2026.1868542)
Supplement: Supplementary file 1 [file Table1.docx]

**Supplementary Material 1**. Search strategy

| **English database search strategy** | | |
| --- | --- | --- |
| **PubMed** | | |
| # | Query | Results |
| 21 | (((("Percutaneous Coronary Intervention"[Mesh]) OR (((Percutaneous Coronary Intervention[Title/Abstract]) OR (Percutaneous Coronary Revascularization[Title/Abstract])) OR (PCI[Title/Abstract]))) AND (((((((((Patient Cooperation[Title/Abstract]) OR (Client Compliance[Title/Abstract])) OR (Client Adherence[Title/Abstract])) OR (Treatment Compliance[Title/Abstract])) OR (Therapeutic Compliance[Title/Abstract])) OR (participat*[Title/Abstract])) OR (complian*[Title/Abstract])) OR (adhere*[Title/Abstract])) OR ("Patient Compliance"[Mesh]))) AND (((("Cardiac Rehabilitation"[Mesh]) OR (((cardiac rehabilitation[Title/Abstract]) OR (Cardiovascular Rehabilitation[Title/Abstract])) OR (rehabilitat*[Title/Abstract]))) OR (("Exercise"[Mesh]) OR (((((((exercise[Title/Abstract]) OR (Physical Activity[Title/Abstract])) OR (Physical Exercise[Title/Abstract])) OR (Acute Exercise[Title/Abstract])) OR (Isometric Exercise[Title/Abstract])) OR (Aerobic Exercise[Title/Abstract])) OR (Exercise Training[Title/Abstract])))) OR ((("Walking"[Mesh]) OR ((Walking[Title/Abstract]) OR (Ambulation[Title/Abstract]))) OR (((((((Cardiorespiratory Fitness[Title/Abstract]) OR (physical fitness[Title/Abstract])) OR (Movement[Title/Abstract])) OR (Motor Activity[Title/Abstract])) OR (strength*[Title/Abstract])) OR (activit*[Title/Abstract])) OR (program*[Title/Abstract]))))) AND (((((((((((meta[Title/Abstract]) OR (meta-analysis[Title/Abstract])) OR (guideline[Title/Abstract])) OR (guide[Title/Abstract])) OR (systematic review[Title/Abstract])) OR (evidence[Title/Abstract])) OR (best evidence[Title/Abstract])) OR (summary[Title/Abstract])) OR (practice guideline[Title/Abstract])) OR (recommendation[Title/Abstract])) OR (consensus[Title/Abstract])) | 170 |
| 20 | ((((((((((meta[Title/Abstract]) OR (meta-analysis[Title/Abstract])) OR (guideline[Title/Abstract])) OR (guide[Title/Abstract])) OR (systematic review[Title/Abstract])) OR (evidence[Title/Abstract])) OR (best evidence[Title/Abstract])) OR (summary[Title/Abstract])) OR (practice guideline[Title/Abstract])) OR (recommendation[Title/Abstract])) OR (consensus[Title/Abstract]) | 3,845,674 |
|  | ((("Cardiac Rehabilitation"[Mesh]) OR (((cardiac rehabilitation[Title/Abstract]) OR (Cardiovascular Rehabilitation[Title/Abstract])) OR (rehabilitat*[Title/Abstract]))) OR (("Exercise"[Mesh]) OR (((((((exercise[Title/Abstract]) OR (Physical Activity[Title/Abstract])) OR (Physical Exercise[Title/Abstract])) OR (Acute Exercise[Title/Abstract])) OR (Isometric Exercise[Title/Abstract])) OR (Aerobic Exercise[Title/Abstract])) OR (Exercise Training[Title/Abstract])))) OR ((("Walking"[Mesh]) OR ((Walking[Title/Abstract]) OR (Ambulation[Title/Abstract]))) OR (((((((Cardiorespiratory Fitness[Title/Abstract]) OR (physical fitness[Title/Abstract])) OR (Movement[Title/Abstract])) OR (Motor Activity[Title/Abstract])) OR (strength*[Title/Abstract])) OR (activit*[Title/Abstract])) OR (program*[Title/Abstract]))) | 6,585,748 |
| 17 | (("Walking"[Mesh]) OR ((Walking[Title/Abstract]) OR (Ambulation[Title/Abstract]))) OR (((((((Cardiorespiratory Fitness[Title/Abstract]) OR (physical fitness[Title/Abstract])) OR (Movement[Title/Abstract])) OR (Motor Activity[Title/Abstract])) OR (strength*[Title/Abstract])) OR (activit*[Title/Abstract])) OR (program*[Title/Abstract])) | 6,178,506 |
| 16 | ((((((Cardiorespiratory Fitness[Title/Abstract]) OR (physical fitness[Title/Abstract])) OR (Movement[Title/Abstract])) OR (Motor Activity[Title/Abstract])) OR (strength*[Title/Abstract])) OR (activit*[Title/Abstract])) OR (program*[Title/Abstract]) | 6,088,124 |
| 15 | ("Walking"[Mesh]) OR ((Walking[Title/Abstract]) OR (Ambulation[Title/Abstract])) | 155,421 |
| 14 | (Walking[Title/Abstract]) OR (Ambulation[Title/Abstract]) | 122,016 |
| 13 | "Walking"[Mesh] | 76,900 |
| 12 | ("Exercise"[Mesh]) OR (((((((exercise[Title/Abstract]) OR (Physical Activity[Title/Abstract])) OR (Physical Exercise[Title/Abstract])) OR (Acute Exercise[Title/Abstract])) OR (Isometric Exercise[Title/Abstract])) OR (Aerobic Exercise[Title/Abstract])) OR (Exercise Training[Title/Abstract])) | 624,201 |
| 11 | ((((((exercise[Title/Abstract]) OR (Physical Activity[Title/Abstract])) OR (Physical Exercise[Title/Abstract])) OR (Acute Exercise[Title/Abstract])) OR (Isometric Exercise[Title/Abstract])) OR (Aerobic Exercise[Title/Abstract])) OR (Exercise Training[Title/Abstract]) | 520,886 |
| 10 | "Exercise"[Mesh] | 285,795 |
| 9 | ("cardiac rehabilitation"[Mesh]) OR (((cardiac rehabilitation[Title/Abstract]) OR (Cardiovascular Rehabilitation[Title/Abstract])) OR (rehabilitat*[Title/Abstract])) | 275,591 |
| 8 | ((cardiac rehabilitation[Title/Abstract]) OR (Cardiovascular Rehabilitation[Title/Abstract])) OR (rehabilitat*[Title/Abstract]) | 275,017 |
| 7 | "cardiac rehabilitation"[Mesh] | 4,730 |
| 6 | ("Patient Compliance"[Mesh]) OR ((((((((Patient Cooperation[Title/Abstract]) OR (Client Compliance[Title/Abstract])) OR (Client Adherence[Title/Abstract])) OR (Treatment Compliance[Title/Abstract])) OR (Therapeutic Compliance[Title/Abstract])) OR (participat*[Title/Abstract])) OR (complian*[Title/Abstract])) OR (adhere*[Title/Abstract])) | 1,288,792 |
| 5 | (((((((Patient Cooperation[Title/Abstract]) OR (Client Compliance[Title/Abstract])) OR (Client Adherence[Title/Abstract])) OR (Treatment Compliance[Title/Abstract])) OR (Therapeutic Compliance[Title/Abstract])) OR (participat*[Title/Abstract])) OR (complian*[Title/Abstract])) OR (adhere*[Title/Abstract]) | 1,256,872 |
| 4 | "Patient Compliance"[Mesh] | 86,374 |
| 3 | ((("Percutaneous Coronary Intervention"[Mesh]) OR (Percutaneous Coronary Intervention[Title/Abstract])) OR (Percutaneous Coronary Revascularization[Title/Abstract])) OR (PCI[Title/Abstract]) | 98,840 |
| 2 | ((Percutaneous Coronary Intervention[Title/Abstract]) OR (Percutaneous Coronary Revascularization[Title/Abstract])) OR (PCI[Title/Abstract]) | 65,021 |
| 1 | "Percutaneous Coronary Intervention"[Mesh] | 71,174 |
| **Web of Science** | | |
| 5 | #1 and #2 and #3 and #4 | 314 |
| 4 | ((((((((((TS=meta) OR (TS=meta-analysis)) OR (TS=guideline)) OR (TS=guide)) OR (TS=systematic review)) OR (TS=evidence)) OR (TS=best evidence)) OR (TS=summary)) OR (TS=practice guideline)) OR (TS=recommendation)) OR (TS=consensus) and Preprint Citation Index (Exclude – Database) | [5,273,546](https://webofscience.clarivate.cn/wos/woscc/summary/d47173cd-43a8-4ee5-824a-d6c8e8d1d9be-0147c92677/relevance/1) |
| 3 | (((((((((((((((((((TS=(Rehabilitation Centers)) OR TS=(cardiac rehabilitation)) OR TS=(Cardiovascular Rehabilitation)) OR TS=(rehabilitat*)) OR TS=(Exercise)) OR TS=(Physical Activity)) OR TS=(Physical Exercise)) OR TS=(Acute Exercise)) OR TS=(Isometric Exercise)) OR TS=(Aerobic Exercise)) OR TS=(Exercise Training)) OR TS=(Walking)) OR TS=(Ambulation)) OR TS=(Cardiorespiratory Fitness)) OR TS=(physical fitness)) OR TS=(Movement)) OR TS=(Motor Activity)) OR TS=(strength*)) OR TS=(activit*)) OR TS=(program*) | 8,149,016 |
| 2 | ((((((((TS=(Patient Compliance)) OR TS=(Patient Cooperation)) OR TS=(Client Compliance)) OR TS=(Client Adherence)) OR TS=(Treatment Compliance)) OR TS=(Therapeutic Compliance)) OR TS=(participat*)) OR TS=(complian*)) OR TS=(adhere*) | 1,398,574 |
| 1 | ((TS=(PCI)) OR TS=(Percutaneous Coronary Intervention)) OR TS=(Percutaneous Coronary Revascularization) | 88,793 |
| **Cochrane Library** | | |
| 22 | #18 and #21 | 81 |
| 21 | #19 or #20 | 257883 |
| 20 | (best evidence):ti,ab,kw OR (summary):ti,ab,kw OR (practice evidence):ti,ab,kw OR (consensus):ti,ab,kw OR (recommendation):ti,ab,kw | 80561 |
| 19 | ("Meta"):ti,ab,kw OR (guideline):ti,ab,kw OR (guide):ti,ab,kw OR (systematic review):ti,ab,kw OR (evidence):ti,ab,kw | 223924 |
| 18 | #15 and #16 and #17 | 302 |
| 17 | #13 or #14 | 266839 |
| 16 | #4 or #5 or #6 or #7 or #8 or #9 or #10 or #11 or #12 | 215787 |
| 15 | #1 or #2 or #3 | 20210 |
| 14 | (participat*):ti,ab,kw OR (complian*):ti,ab,kw OR (adhere*):ti,ab,kw | 266774 |
| 13 | MeSH descriptor: [Patient Compliance] explode all trees | 15722 |
| 12 | MeSH descriptor: [Rehabilitation] explode all trees | 56928 |
| 11 | ((exercise* or fitness) near/3 (treatment or intervent* or program*)):ti,ab,kw | 37436 |
| 10 | (train*) near (strength* or aerobic* or exercise*):ti,ab,kw | 35828 |
| 9 | MeSH descriptor: [Exercise] explode all trees | 40288 |
| 8 | (physical* near (fit* or train* or therap* or activit*)):ti,ab,kw | 80153 |
| 7 | rehabilitat*:ti,ab,kw | 75023 |
| 6 | MeSH descriptor: [Physical Exertion] explode all trees | 4884 |
| 5 | MeSH descriptor: [Sports] explode all trees | 22841 |
| 4 | MeSH descriptor: [Exercise Therapy] explode all trees | 22886 |
| 3 | pci or ptca | 12760 |
| 2 | (percutaneous next coronary near/2 (interven* or revascular*)) | 13951 |
| 1 | MeSH descriptor: [Percutaneous Coronary Intervention] explode all trees | 9038 |
| **Embase** | | |
| 6 | #1 and #2 and #3 and #4 | 426 |
| 4 | 'systematic review'/exp OR 'meta analysis'/exp OR 'systematic review':ti,ab,kw OR 'meta-analysis':ti,ab,kw OR 'meta analysis':ti,ab,kw OR guideline*:ti,ab,kw OR 'clinical guideline':ti,ab,kw OR 'practice guideline':ti,ab,kw OR consensus:ti,ab,kw OR 'expert consensus':ti,ab,kw OR 'evidence summary':ti,ab,kw OR 'best evidence':ti,ab,kw OR 'best evidence summary':ti,ab,kw OR 'evidence synthesis':ti,ab,kw OR 'randomized controlled trial'/exp OR 'controlled clinical trial'/exp OR random*:ti,ab,kw OR trial:ti,ab,kw OR 'quasi-experimental':ti,ab,kw OR 'quasi experimental':ti,ab,kw OR 'controlled study':ti,ab,kw OR 'prospective controlled study':ti,ab,kw | 5,266,882 |
| 3 | 'patient compliance'/exp OR adheren*:ti,ab,kw OR complian*:ti,ab,kw OR attendance:ti,ab,kw OR uptake:ti,ab,kw OR participation:ti,ab,kw OR completion:ti,ab,kw OR dropout*:ti,ab,kw OR attrition:ti,ab,kw OR utilization:ti,ab,kw OR utilisation:ti,ab,kw OR enrolment:ti,ab,kw OR enrollment:ti,ab,kw OR engagement:ti,ab,kw OR nonattendance:ti,ab,kw OR 'non-attendance':ti,ab,kw | 2,739,164 |
| 2 | 'cardiac rehabilitation'/exp OR 'cardiac rehabilitation':ti,ab,kw OR 'cardiovascular rehabilitation':ti,ab,kw OR 'heart rehabilitation':ti,ab,kw OR 'exercise-based cardiac rehabilitation':ti,ab,kw OR 'exercise based cardiac rehabilitation':ti,ab,kw OR 'exercise rehabilitation':ti,ab,kw | 26,836 |
| 1 | 'percutaneous coronary intervention'/exp OR 'percutaneous coronary intervention':ti,ab,kw OR pci:ti,ab,kw OR ptca:ti,ab,kw OR 'coronary angioplasty':ti,ab,kw OR 'coronary stent*':ti,ab,kw OR 'post pci':ti,ab,kw OR 'after pci':ti,ab,kw | 205,823 |
| **Wiley** | | |
| 1 | "adherence" in Title and "PCI" in Title and "cardiac rehabilitation" in Title | 0 |
| **UpToDate** | | |
| 1 | cardiac rehabilitation AND exercise | 10 |
| **BMJ Best practice(BMJ)** | | |
| 1 | adherence to exercise-based cardiac rehabilitation | 65 |
| **the Joanna Briggs Institute(JBI)** | | |
| 1 | adherence to exercise-based cardiac rehabilitation | 13 |
| **the Guidelines International Network(GIN)** | | |
| 1 | Cardiac rehabilitation | 6 |
| **the Scottish Intercollegiate Guidelines Network(SIGN)** | | |
| 1 | adherence to exercise-based cardiac rehabilitation | 17 |
| **the National Institute for Health and Care Excellence(NICE)** | | |
| 1 | adherence to exercise-based cardiac rehabilitation | 14 |
| **the European Society of Cardiology(ESC)** | | |
| 1 | adherence to exercise-based cardiac rehabilitation | 30 |
| **the American Heart Association(AHA)** | | |
| 1 | adherence to exercise-based cardiac rehabilitation | 289 |
| **American College of Cardiology （ACC）** | | |
| 1 | adherence to exercise-based cardiac rehabilitation | 201 |

| **Chinese database search strategy** | | |
| --- | --- | --- |
| **CBM** | | |
| 1 | ("经皮冠状动脉介入治疗"[常用字段:智能] OR "冠脉支架植入术"[常用字段:智能] OR "PCI"[常用字段:智能] OR "经皮冠状动脉治疗"[常用字段:智能] OR "经皮冠状动脉介入术"[常用字段:智能]) OR ("经皮冠状动脉介入治疗"[不加权:扩展]) | 53621 |
| 2 | ("心脏康复"[常用字段:智能] OR "康复"[常用字段:智能] OR "运动"[常用字段:智能] OR "锻炼"[常用字段:智能] OR "训练"[常用字段:智能] OR "活动"[常用字段:智能]) OR ("心脏康复"[不加权:扩展]) | 1250495 |
| 3 | ("病人依从"[常用字段:智能] OR "依从性"[常用字段:智能] OR "健康行为"[常用字段:智能] OR "遵守"[常用字段:智能] OR "遵医"[常用字段:智能]) OR ("病人依从"[不加权:扩展]) | 163801 |
| 4 | "Meta分析"[常用字段:智能] OR "指南"[常用字段:智能] OR "证据"[常用字段:智能] OR "最佳证据"[常用字段:智能] OR "系统评价"[常用字段:智能] OR "循证实践"[常用字段:智能] OR "规范"[常用字段:智能] OR "共识"[常用字段:智能] OR "证据总结"[常用字段:智能] | 493143 |
| 5 | (#4) AND (#3) AND (#2) AND (#1) | 44 |
| **CNKI** | | |
| 1 | (SU=经皮冠状动脉介入治疗 + 经皮冠状动脉介入术 + 经皮冠状动脉治疗 + PCI + 冠脉支架植入术 + PTCA) |  |
| 2 | (SU=心脏康复 + 康复 + 运动 + 锻炼 + 训练 + 活动) |  |
| 3 | (SU=病人依从 + 依从性 + 健康行为 + 遵守 + 遵医) |  |
| 4 | (SU=指南 + 证据 + 最佳实践 + 系统评价 + 循证实践 + 规范 + 共识 + 证据总结 + Meta分析) |  |
| 5 | #1 AND #2 AND #3 AND #4 | 23 |
| **VIP** | | |
| 1 | ((((((((题名或关键词=经皮冠状动脉介入治疗 OR 题名或关键词=经皮冠状动脉介入术) OR 题名或关键词=经皮冠状动脉治疗) OR 题名或关键词=PCI) OR 题名或关键词=冠脉支架植入术) OR 题名或关键词=PTCA) AND (((((题名或关键词=心脏康复 OR 题名或关键词=康复) OR 题名或关键词=运动) OR 题名或关键词=锻炼) OR 题名或关键词=训练) OR 题名或关键词=活动)) AND ((((题名或关键词=病人依从 OR 题名或关键词=依从性) OR 题名或关键词=健康行为) OR 题名或关键词=遵守) OR 题名或关键词=遵医)) AND ((((((((题名或关键词=指南 OR 题名或关键词=证据) OR 题名或关键词=最佳实践) OR 题名或关键词=系统评价) OR 题名或关键词=循证实践) OR 题名或关键词=规范) OR 题名或关键词=共识) OR 题名或关键词=证据总结) OR 题名或关键词=Meta分析)) | 0 |
| **wanfang** | | |
| 1 | 主题:(经皮冠状动脉介入治疗 or 经皮冠状动脉介入术 or 经皮冠状动脉治疗 or PCI or 冠脉支架植入术 or PTCA) and (心脏康复 or 康复 or 运动 or 锻炼 or 训练 or 活动) and (病人依从 or 依从性 or 健康行为 or 遵守 or 遵医) and (指南 or 证据 or 最佳实践 or 系统评价 or 循证实践 or 规范 or 共识 or 证据总结 or Meta分析) | 113 |
| **Yimaitong** | | |
| 1 | PCI术后基于运动的心脏康复依从性 | 58 |
